# Accurate Diagnosis of *Schistosoma mansoni* and *S. haematobium* from Filtered Urine Samples Collected in Tanzania, Africa

**DOI:** 10.3390/pathogens13010059

**Published:** 2024-01-08

**Authors:** Koreena Miller, Javeriya Choudry, El Shaimaa Mahmoud, Nilanjan Lodh

**Affiliations:** Department of Medical Laboratory Science, Marquette University, Milwaukee, WI 53217, USA; koreena.miller@marquette.edu (K.M.); javeriya.choudry@marquette.edu (J.C.); e.mahmoud96@outlook.com (E.S.M.)

**Keywords:** *Schistosoma mansoni*, *S. haematobium*, urine, molecular diagnosis

## Abstract

Schistosomiasis is a bloodborne, and waterborne parasitic disease caused by the human *Schistosoma* species, namely *Schistosoma mansoni* and *S. haematobium*. The parasite requires an intermediate snail host, where they grow and develop, along with a human host (definitive). *Schistosoma* egg detection in feces (*S. mansoni*) and urine (*S. haematobium*) are the WHO-recommended confirmatory diagnostic tests. The goal of our research was to determine the efficacy of detecting single or dual Schistosome species from filtered human urine samples collected in Tanzania by amplifying species-specific cell-free repeat DNA fragments via polymerase chain reaction (PCR) and gel electrophoresis. In total, 104 filtered human urine samples were evaluated and collected from individuals residing in the village of Kayenze, Tanzania. All samples were detected with 100% accuracy and no cross-amplification was present. For a single infection of *S. mansoni*, 22 (21%) of the samples were positive, while 15 (14%) of the samples were negative via PCR. Moreover, for a single infection of *S. haematobium*, 7 (7%) of the samples were positive, while 15 (14%) of the samples were negative. Dual infections were found in a higher percentage, with 60 (58%) of the samples being positive. Thus, we have justified that PCR is more sensitive and specific by amplifying species-specific cell-free repeat DNA fragments from the same urine sample than WHO-recommended methods of processing stool and urine.

## 1. Introduction

The World Health Organization (WHO) estimates that at least 90% of people requiring treatment for schistosomiasis live in Africa [1]. After malaria, schistosomiasis is the biggest cause of morbidity in sub-Saharan Africa. This is a blood-borne and water-borne parasitic disease prevalent in poor and rural communities, specifically fishing and agricultural populations. There are two primary types of human Schistosomes in Africa: intestinal disease caused by *Schistosoma mansoni*, and urogenital disease caused by *S. haematobium*. In Tanzania, it is estimated that over 23 million people (51.5% of people) are infected with schistosomiasis [2]. In 2019, between 5% and 9.9% of the global population requiring preventative chemotherapy for schistosomiasis were in Tanzania [3].

Currently, WHO recommends *S. mansoni* to be detected via the presence of eggs in fecal specimens through Kato–Katz (KK) or detecting circulating cathodic antigen (CCA) and *S. haematobium* to be detected based on the presence of eggs through a filtration technique or chemical reagent strips for hematuria (blood in urine) [3]. KK, alongside the use of microscopy, has 100% specificity but this method is not sensitive enough to detect low-level infections. While CCA and chemical reagent strips are more sensitive than microscopy, they are not sensitive enough for low-level infection [4]. Additionally, these tests lack specificity [4].

With the use of mass drug administration (MDA) for the treatment of schistosomiasis, low levels of infections must be detected. Detection is a key tool for mapping and transmission assessment [3]. It will be useful to determine the effectiveness of MDA and monitor possible future resistance to the only drug, i.e., praziquantel (PZQ), that treats all three major human Schistosome species. Additionally, even with preventative MDA, there is still a possibility of low-level infection. These low-level infections must be detected to prevent transmission and provide individuals with further treatment. 

We have developed and used a schistosome diagnostic test that is highly sensitive and specific. This test involves filtering 20–30 mL of urine through filter paper (Whatman # 3) and transporting the dried paper to a lab where DNA can be extracted from the paper, after which species-specific repeat fragments can be amplified via PCR, and the amplification can be visualized by gel electrophoresis to determine the presenting infection [5]. In previous geographic locations, such as Ghana and Zambia, we demonstrated that it is possible to detect DNA specific to both *S. mansoni* and *S. haematobium* from a single source of urine, simplifying the collection process and performance of tests [5,6]. This method is more sensitive and more specific than standard diagnostic tests [5,6,7]. 

The test relies upon the use of cell-free repeat DNA biomarkers. Detection of cell-free repeat DNA is viable, more sensitive, and a more effective biomarker than a gene-based marker [7,8]. The repeat fragments are present in a high number in the genome (sometimes more than 1000 per haploid genome), mostly as non-coding fragments with no protein-coding function, and evolve faster than the rest of the genome, which enables high detection sensitivity and specificity [8]. We have demonstrated the presence of helminth and protozoan parasite species-specific DNA in urine in the past, such as *S. mansoni* [9], *S. haematobium* [10], *Strongyloides stercoralis* [11], and *Plasmodium falciparum* [12]. 

For *S. mansoni*, the cell-free repeat DNA sequence, Sm1-7 (GenBank: M61098.1), is a 121 base pair non-coding sequence [13]. For *S. haematobium*, the cell-free repeat DNA sequence, Dra1 (GenBank: DQ157698.1), is also a 121 base pair non-coding sequence [9]. For both schistosome species, the repeat fragments comprised 12–16% of each parasite’s genome (~600,000 copies per cell), were species-specific, and occurred in different regions of genomes of these two schistosome parasites, so there is no chance of cross-amplification [14]. In addition, the non-coding nature of the repeat segments allows them to be less likely to be mutated as the schistosome parasite undergoes genetic changes.

We aim to determine the efficacy of detecting dual Schistosome parasites from filtered human urine samples collected in Tanzania by amplifying species-specific cell-free repeat DNA fragments via polymerase chain reaction (PCR). The use of PCR will allow for the detection of low-level infections with fewer false negatives. 

## 2. Materials and Methods

### 2.1. Sample Location and Sample Population 

The study was conducted in the Kayenze village in Tanzania. People living in the village were chosen for this study because this was a fishing community and schistosomiasis infection was highly prevalent. There was a total of 350 participants examined for schistosomiasis and HIV, of which 200 were women and 150 were men. All participants were adults due to the collection of blood samples. A total of 104 samples were analyzed in the United States for the presence or absence of dual schistosome species.

### 2.2. Ethical Approval 

The Institutional Review Board (IRB # NIMR/HQ/R.8a/Vol. IX/2679) approval was acquired from the Ministry of Health, Dodoma, Tanzania, to conduct the study. The Material Transfer Agreement (MTA) was established for sample transfer between Medical Mission Institute Wurzburg, Germany, and Marquette University, USA. A Certification Statement was established for both institutions in Germany and the USA for sample clearance through the United States Customs and reaching the laboratory in the USA for evaluation. 

### 2.3. Sample Collection

Urine, serum, and plasma samples were collected for evaluation via parasitological, immunological, and molecular testing. Serum and plasma samples were collected before Praziquantel (PZQ) treatment of patients and then again five to nine days after treatment. Urine filter papers were prepared from 104 urine samples five to nine days after PZQ treatment. To prepare the filters, 6–20 mL of urine was actively pressed with a syringe through a cone-shaped 2 cm Whatman No. 3 (Whatman International, Maidstone, UK) filter paper. The filter paper was then marked with patient identification. The filter papers were left to air dry under a fly-proof bed net to prevent any external contamination and then packed with desiccant in individual, sealed Ziplock bags. The urine samples were then shipped to Marquette University, Wisconsin, USA, for DNA extraction and molecular diagnosis.

### 2.4. KK and CCA Test 

The Kato–Katz (KK) kit, a WHO-recommended kit (WHO, Geneva, Switzerland), was used to detect the presence of *S. mansoni* eggs [15]. Briefly, feces were pressed through a mesh screen to remove large particles. A portion of the sieved stool sample was transferred to the hole of a template placed on a slide. After filling the hole on the template, the template was removed, and the remaining sample was covered with a piece of cellophane previously soaked in glycerol-malachite green. The slide was then examined under a microscope for the presence of *S. mansoni* eggs.

The presence of *S. mansoni* infections was also tested with the circulating cathodic antigen (CCA) rapid test (Rapid Medical Diagnostics, Pretoria, South Africa) based on the manufacturer’s recommendation. One drop of urine was added into the cassette well and a drop of the reagent buffer was added and left undisturbed for 20 min. Two results were possible (either positive or negative). For a positive result, both the control band and the test band appear red, while, in a negative test, only the control line appears red. 

### 2.5. Hematuria Test 

Microhematuria (blood in urine) was detected in nine samples. All these samples were tested for *S. haematobium* eggs via urine microscopy. 

### 2.6. DNA Extraction 

One-quarter of the round filter paper was cut by a scissor and punched with a hole puncher. In between each sample, the hole puncher and scissor were cleaned with 10% bleach and water and dried to prevent any cross-contamination. The punches from each sample were placed into 2 mL Eppendorf tubes containing 600 μL nuclease-free water. The tubes were incubated at 95 °C for 10 min and then placed on a rotator at room temperature overnight (16–18 h). Then, the water containing the DNA was transferred to Qiagen QIAmp 2 mL column tubes. The DNA was precipitated and concentrated using the QIAmp DNA Blood Mini Kit according to the manufacturer’s protocol. In summary, the collection tubes were first centrifuged at 8000 rpm for 1 min. After discarding the liquid, 500 μL of AW1 wash buffer was added to the collection tubes and left to stand for 5 min. After centrifuging at 8000 rpm for 1 min, the liquid was discarded. Subsequently, 500 μL of AW2 wash buffer was added to the collection tubes and the tubes were centrifuged at 14,000 rpm for 3 min. The liquid was discarded, and the collection tubes were centrifuged at maximum speed for 2 min. The columns were then transferred to 1.5 mL tubes and the collection tubes were discarded. Following this, 200 μL AE elution buffer was added to the tubes. The tubes were left to stand for 2 min and were then centrifuged at 8000 rpm for 3 min. The DNA concentration for each sample was measured via a NanoDrop ND-1000 spectrophotometer (NanoDrop Technologies, Wilmington, DE, USA). Lastly, aliquots were prepared, and both the stock and aliquots were stored for future use at −20 °C.

### 2.7. PCR and Gel Electrophoresis

PCR was administered on all 104 samples to detect the presence of *S. mansoni* (121 bp), *S. haematobium* (121 bp), or both species. Species-specific primers (Table 1) were used to amplify repeat DNA fragments from extracted DNA. Both primers were designed (Integrated DNA Technologies: IDT, Coralville, IA) based on the 121 bp Sm1-7 repeat fragment of *S. mansoni* (GenBank: M61098.1) and 121 bp Dra1 repeat fragment of *S. haematobium* (GenBank: DQ157698.1). For every reaction, *S*. *mansoni* and *S*. *haematobium* genomic DNA (BEI Resources, Manassas, VA, USA) were used as a positive control, extracted DNA from urine collected in-house was used as a negative control, and nuclease-free-water (Sigma-Aldrich, St. Louis, MO, USA) was used as water control. For amplification, 2 μL of DNA served as a PCR template. The total PCR reaction volume was 10 μL and consisted of 5 μL of PCR Master Mix (New England Biolabs, Ipswich, MA, USA), 0.5 μL of each of the amplification primers (forward and reverse), 0.5 μL of 25 mM MgCl_2_, and 2 μL of nuclease-free-water. PCR amplification took place in an automated thermocycler. Amplification of each sample was done twice with each set of primers. If there were any ambiguous results, the reaction was repeated a third time. If there was any contamination issue related to negative and water controls coming out as positives, then all the results from the sample run were discarded and PCR was redone for that entire sample run. All samples were identified with 100% specificity without any cross-amplification.

For amplification of *S. mansoni*, initial denaturation occurred at 95 °C for 10 min, which was followed by 35 cycles of denaturation at 95 °C for 30 s, then annealing at 65 °C for 90 s, extension at 72 °C for 1 min, and a final extension at 72 °C for 10 min. For amplification of *S. haematobium*, initial denaturation occurred at 95 °C for 10 min, which was followed by 35 cycles of denaturation at 95 °C for 30 s, then annealing at 63 °C for 90 s, extension at 72 °C for 1 min, and a final extension at 72 °C for 5 min. The electrophoresis of 4 μL of amplified PCR product was carried out on a 2% agarose gel stained with SYBR Green (Thermo Scientific, Waltham, MA, USA). A 50 bp reference ladder (New England BioLabs Inc., Ipswich, MA, USA) was used to estimate amplified band sizes. All agarose gels were visualized in the Azure C200 gel documentation system (Azure Biosystems, Dublin, CA, USA). 

### 2.8. Statistical Analysis

The data were stratified for three different age groups (Group A: 17–30 years, Group B: 31–50 years, and Group C: 51–97 years) and sex (female and male). The results were converted to numerical values (1 = positive and 0 = negative) for performing the statistical analysis. The positive and negative assessment of each sample was based on the following predictions of duplicate runs for all samples.

- If KK +, then there is surely the presence of *S. mansoni* infection.
- If *S. mansoni* PCR +, then we assume the presence of *S. mansoni* infection.
- *S. mansoni* true positive (TP): KK + and *S. mansoni* PCR +
- *S. mansoni* true negative (TN): KK − and *S. mansoni* PCR −
- *S. haematobium* true positive (TP): *S. haematobium* PCR +
- *S. haematobium* true negative (TN): *S. haematobium* PCR −

PCR efficacy, single vs. dual infection, gender-specific, and age group-specific analyses were performed using JMP (v9, SAS Institute Inc., Cary, NC, USA). PCR efficacy was also determined against KK and CCA for *S. mansoni*. Disease prevalence was calculated based on the number of positive cases by each diagnostic test against the total number of samples that were evaluated. Sex-specific and age group (17–30 years, 31–50 years, and 51–97 years)-specific evaluations (JMP v9, SAS Institute Inc.) of positivity and negativity of each diagnostic test were also conducted to establish the robustness of the PCR test. The sensitivity and specificity of PCR amplification for *S. mansoni* were compared against KK and CCA using MedCalc 12.4.0 (MedCalc Software, Ostend, Belgium). 

## 3. Results

### 3.1. Detection of Dual Schistosome Parasite Infection 

**Figure 1:**
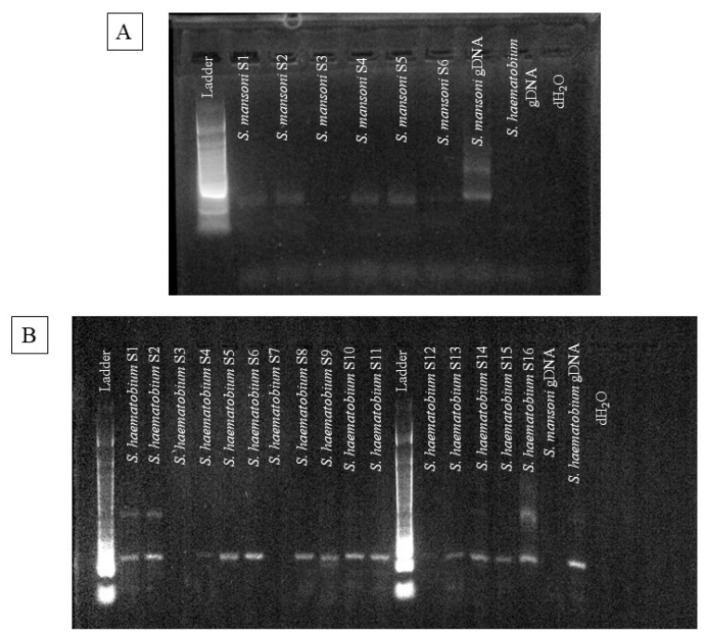
Agarose gel images of the amplified repeat fragment from field-collected filtered urine samples from Tanzania. (**A**). Repeat fragment amplification by PCR for *Schistosoma mansoni*. (**B**). Repeat fragment amplification by PCR for *S. haematobium*. Abbreviations: Ladder = reference DNA; S = Sample; gDNA = genomic DNA; dH2O = DNA-RNA free water.

PCR was performed to detect both *S. mansoni* and *S. haematobium* for single or dual infection with high sensitivity and specificity (Figure 1). A total of 104 samples were amplified via PCR to detect both species from the DNA extracted from the same filtered urine samples. For a single infection of *S. mansoni*, 22 (21%) of the samples were positive, while 15 (14%) of the samples were negative via PCR amplification. For a single infection of *S. haematobium*, 7 (7%) of the samples were positive, while 15 (14%) of the samples were negative. *S. mansoni* and *S. haematobium* were found in higher percentages as co-infections, with 60 (58%) of the samples presenting as co-infections (Table 2). 

### 3.2. Prevalence Data for S. mansoni 

*S. mansoni* infections were detected at a higher rate with 82 positives (79%, Table 3). In total, 22 of the samples were negative for *S. mansoni* (Table 3). Within the three age groups tested for disease prevalence, *S. mansoni* was the most prevalent at 35% in the age group of 31–50 years (highest), then at 25% among the age group of 17–30 years, and 19% among the age group of 51 to 97 years (Table 4). *S. mansoni* was present at a higher density in females (52% compared to 27% in males) (Table 4).

### 3.3. Prevalence Data for S. haematobium 

*S. haematobium* was detected at a moderate level in all the evaluated filtered urine samples via PCR. There were 67 positives (64%) and 37 negatives (36%) for *S. haematobium* out of all the samples infected with Schistosomiasis (Table 2). *S. haematobium* was also present at a higher density among females (41%) compared to males (23%). Out of all three age groups evaluated, the 31–50 years age group had the highest prevalence of *S. haematobium* infection compared to other age groups (Table 4). 

### 3.4. Comparison of S. mansoni Detection Methods 

Along with PCR, microscopy via Kato–Katz (KK) and POC-CCA (CCA) was conducted to detect *S. mansoni* infection. There were more true positives via PCR than CCA. Out of the total samples, 64 (61%) were positive for *S. mansoni* via both KK and CCA and 78 (75%) of the samples were positive for *S. mansoni* by both KK and PCR. There were fewer false negatives for PCR than CCA. While 5 (5%) of the samples were negative via KK and positive via CCA, 4 (4%) of the samples were negative via KK and positive via PCR. There were also fewer true negatives for PCR than CCA. Additionally, 35 (34%) of the samples were positive via KK and negative via CCA, while 21 (20%) of samples were positive via KK and negative via PCR. PCR resulted in greater detection of true negatives than CCA. Also, while 0 samples were negative via KK and CCA, there was 1 (1%) that was negative via both KK and PCR (Table 5).

### 3.5. Comparison of S. haematobium Detection Methods

Urine microscopy was performed for *S. haematobium* on 9 samples which were positive for microhaematuria. Urine microscopy was negative for all the samples and did not detect any *S. haematobium* eggs. Moreover, 7 (78%) out of 9 samples were positive for PCR and 2 were negative. 

### 3.6. Diagnostic Parametrs for S. masoni

For *S. mansoni*, the sensitivity of KK (95.2%) was higher compared to CCA (66.4%) and PCR (80%). Specificity for all three tests was at 100%. *S. haematobium* parameters were not calculated as urine microscopy was performed on only 9 microhaematuria-positive samples (Table 6). 

## 4. Discussion

We have shown that PCR detection of both schistosome species is more sensitive and specific by amplifying species-specific cell-free repeat DNA fragments from the same urine sample rather than processing stool and urine. Schistosome species-specific repeat DNA fragments allow differentiation between *Schistosoma* species without any cross-amplification. 

The diagnostic problem for schistosomiasis is exacerbated by the fact that in Africa two major human species, *S. mansoni* and *S. haematobium*, are often sympatric, concurrent infection is debilitating, and common and current measures lack sensitivity and specificity [16]. Additionally, the concurrent nature of the two species raises the problem of an accurate and specific diagnosis, especially when infections are asymptomatic and of low intensity during control programs, especially after MDA [17]. The prevalence of *S. mansoni* is predominant (79%, Table 3) in our current findings, although the chances of having a concurrent infection are higher, with 58% exhibiting concurrent infections for the samples evaluated from Tanzania (Table 2). Currently, clinical and epidemiological studies are often unable to detect low-level infections as well as sympatric, multiple parasite infections due to a lack of capacity, sensitivity, and specificity of current parasitological and proposed immunological techniques [18]. The Kato–Katz technique (KK, WHO recommended) and circulating cathodic antigen test (CCA) for *S. mansoni* and urine filtration for *S. haematobium* lack sensitivity, and hematuria (blood in urine) for *S. haematobium* lacks specificity. These procedures involve examining both stool and urine for eggs, and blood and urine for the presence of parasite antigens or antibodies. Logistically and technically, such tasks are time-consuming and inadequately sensitive, and they often produce false negative results as antibodies persist even after successful treatment [9,10]. Addressing these diagnostic difficulties is especially important as many African countries, such as Tanzania are moving towards a goal of eliminating schistosomiasis through mass drug administration (MDA), which lowers infection prevalence. Evaluation of the PCR test for Schistosome species from field-collected urine samples across age groups is extremely important as it significantly increases the accuracy of the diagnosis of both species. Our unique approach of sample collection, transport, and storage for PCR tests is novel and there is no need to collect and process stool, whole urine, or whole blood to scrutinize them for eggs. 

The current study evaluated all the individuals who were treated with praziquantel (PZQ), and all urine samples were collected on average 5–7 days after the PZQ treatment (data not shown). There is still a significant remaining, recurring, or trace infection remaining and detected by species-specific cell-free DNA detection. Additionally, it might not be feasible to treat the entire population. Targeted treatment of individuals or demographic groups who remain persistently infected [19] is more effective and prevents any future resistance to the only drug (i.e., PZQ) commonly used to treat all three major human schistosome species, including *S. japonicum* [20].

Schistosomiasis is also strongly related to the age of the person infected: in children aged 6–15, the prevalence of the infection and its intensity peaks, while, for older adults, the prevalence decreases. Because of this discrepancy between age groups, the WHO recommends diagnostic tests with high sensitivity, and it also tracks the efficacy of control efforts [16,21,22]. In our current findings, infections are more prevalent in females (*S. mansoni*: 52% and *S. haematobium*: 41%) than males (*S. mansoni*: 27% and *S. haematobium*: 23%) for both species (Table 4). The infection level is also higher for the middle age group (31–50 years) for Schistosome species compared to the younger (17–30 years) and older (51–97 years) age groups (Table 5). 

The small sample size of the study might be a limiting factor when evaluating the full scope of the diagnostic and control efficacy. The PCR test will be implemented in larger clinical settings in the future. This will allow us to evaluate the performance of our assay against other WHO-recommended tests for both Schistosome species. PCR detected more positive infections compared to CCA. Some urine samples came out negative by PCR, although these were positive by KK (Table 5). This might be because KK and CCA were performed before the MDA treatment of the participating individuals. Urine samples were also collected on average 5–7 days after the MDA treatment with PZQ. The treatment might have resolved the infection and cleared the parasites from the infected individuals. Also, no residual, remaining, or new infections occurred over the 5–7 days. So, the Schistosome DNA was absent in the collected urine samples. In return, PCR did not detect any positive infection among those urine samples (21, Table 5), which are positive for KK, although there were four negative KK samples detected as positive by PCR. 

Accurate diagnosis of schistosomiasis is also particularly important as both species affect different body systems and can cause concurrent infections. To evaluate the success of control programs, to conduct surveillance, and to determine the prevalence of dual schistosome infection for moderate and low infection areas, a more sensitive, specific, quick, and cost-effective test is needed. We have evaluated and demonstrated the need for a more accurate and sensitive testing method to identify infected patients.

## Figures and Tables

**Table 1:** Schistosome species-specific primers are used to amplify repeat cell-free DNA fragments from extracted DNA.

Schistosome Parasite	Oligonucleotide Name	Oligonucleotide Sequence
*Schistosoma mansoni*	SmPF	5′ GAT CTG AAT CCG ACC AAC CG 3′
	SmPR	5′ ATA TTA ACG CCC ACG CTC TC 3’
*Schistosoma haematobium*	ShDra1F	5′ TCA CAA CGA TAC GAC CAA C 3′
	ShDra1R	5′ GAT CTC ACC TAT CAG ACG AAA C 3′

**Table 2:** Comparative analysis of single and dual Schistosome infection.

	*S. mansoni* PCR			
***S. haematobium PCR***	**Count**	**0**	**1**	**Total**
	**0**	15 (14%)	22 (21%)	37
	**1**	7 (7%)	**60 (58%)**	67
	**Total**	22	82	104

**Table 3:** PCR results for *S. mansoni* and *S. haematobium* individually.

Species	PCR +	PCR −
*S. mansoni*	82 (79%)	22 (21%)
*S. haematobium*	67 (64%)	37 (36%)

**Table 4:** Comprehensive analysis of Schistosome infection for both genders and three age groups.

Schistosome Species	Gender		Age Group		
	Male	Female	A (17–30 yrs)	B (31–50 yrs)	C (51–97 yrs)
*S. mansoni*	28 (27%)	54 (52%)	26 (25%)	36 (35%)	20 (19%)
*S. haematobium*	24 (23%)	43 (41%)	20 (19%)	29 (28%)	18 (17%)

**Table 5:** Comprehensive analysis of *Schistosoma mansoni* infection detected by Kato–Katz (KK), circulating cathodic antigen (CCA), and polymerase chain reaction (PCR).

Diagnostic Tests	CCA		PCR	
	Positive	Negative	Positive	Negative
KK Positive	64 (61%)	35 (34%)	78 (75%)	21 (20%)
KK Negative	5 (5%)	0	4 (4%)	1 (1%)

**Table 6:** Diagnostic parameter evaluation for KK, CCA, and PCR for *S. mansoni*. *S. haematobium* was not included as urine microscopy was performed on only 9 microhaematuria-positive samples.

Diagnostic Test Parameters	Kato–Katz (*S. mansoni*)	CCA (*S. mansoni*)	PCR (*S. mansoni*)
Sensitivity	95.2%	66.4%	80%
Specificity	100%	100%	100%

## Data Availability

Data is available and will be provided upon request.
